# BePLi Dataset v1: Beach Plastic Litter Dataset version 1 for instance segmentation of beach plastic litter

**DOI:** 10.1016/j.dib.2023.109176

**Published:** 2023-04-23

**Authors:** Mitsuko Hidaka, Koshiro Murakami, Kenta Koshidawa, Shintaro Kawahara, Daisuke Sugiyama, Shin'ichiro Kako, Daisuke Matsuoka

**Affiliations:** a Research Institute for Value-Added-Information Generation (VAiG), Japan Agency for Marine-Earth Science and Technology (JAMSTEC), Kanagawa, Japan; b Graduate School of Science and Engineering, Kagoshima University, Kagoshima, Japan

**Keywords:** Coastal litter, Marine plastics, AI, Deep learning, Object detection, Beach monitoring

## Abstract

Marine plastic pollution is a pressing global issue nowadays. To address this problem, automated image analysis techniques that can identify plastic litter are necessary for scientific research and coastal management purposes. The Beach Plastic Litter Dataset version 1 (BePLi Dataset v1) comprises 3709 original images taken in various coastal environments, along with instance-based and pixel-level annotations for all plastic litter objects visible in the images. The annotations were compiled in the Microsoft Common Objects in Context (MS COCO) format, which was partially modified from the original format. The dataset enables the development of machine-learning models for instance-level and/or pixel-wise identification of beach plastic litter. All original images in the dataset were extracted from beach litter monitoring records operated by the local government of Yamagata Prefecture in Japan. Litter images were taken in different backgrounds, such as sand beaches, rocky beaches, and tetrapods. The annotations for instance segmentation of beach plastic litter were made manually, and were given for all plastics objects, including PET bottles, containers, fishing gear, and styrene foams,all of which were categorized in a single class “plastic litter”. Technologies developed using this dataset have the potential to enable further scalability for the estimation of plastic litter volume. This would help researchers, including individuals, and the the government to monitor or analyze beach litter and the corresponding pollution levels.

**Specifications Table**

**Table:** 

Subject:	Pollution, Nature and Landscape Conservation, Computer Vision and Pattern Recognition, Artificial Intelligence, Computer Science
Specific subject area:	Instance-based or pixel-level image classification or both for detecting beach plastic litter
Type of data:	PNG original images, JSON files for the object detection task compiled in Microsoft Common Objects in Context (MS COCO) [1] (deliberately modified in part)
How the data were acquired:	The original images were captured by various observers using standard consumer digital cameras. Image resolutions and PNG data compression ratios were inconsistent across different digital cameras. Instance segmentation masks were created over the original images using Adobe Photoshop (Adobe Inc.). Deliberately modified MS COCO-formatted JSON files were created from the instance segmentation mask using Python 3.9 and compatible libraries (such as Pycocotools 2.0 and Pillow 8.4).
Data format:	Raw, Annotated, Converted
Description of data collection:	Pictures of beach litter were obtained from coastal areas in spring and autumn every year from 2011 to 2019 to monitor the degree of pollution on the beaches in Yamagata Prefecture. The pictures were taken from three (front, left, and right) or four (front, left, right, and back) directions from the observer; extra close-up shots of litter were also taken.
Data source location:	**Institution:** Yamagata Prefecture Government **City/Town/Region:** Yamagata Prefecture **Country:** Japan Images from the entire coastal area in the Yamagata Prefecture were used.
Data accessibility:	**Repository name:** Sea Open Scientific Data Publication (SEANOE) **Data identification number:** 92297 **Direct URL to data:**https://doi.org/10.17882/92297

## Value of the Data


 

• Marine plastic pollution is a major global issue nowadays. To address this problem, it is important to determine the amount of plastic litter in the natural environment for scientific research and management purposes. Automated image analysis techniques for identifying plastic litter are essential for quantification, but training datasets for machine learning must be first obtained to develop such technologies.
• Accessible datasets for the segmentation of beach plastic litter are extremely rare. This dataset is unique in that it was created based on images depicting litter in its natural state, which makes it very practical. Furthermore, the dataset is valuable because its manually generated high-quality pixel-level annotations are more expensive than those created for image classification or bounding box-based object detection.
• This dataset can be used to develop machine learning technologies to detect beach plastic litter at the pixel level, which has the potential to enable further scalability for the estimation of plastic litter volume. These technologies can assist researchers and local communities, including the local government, in monitoring and analyzing beach litter and pollution levels.
• Additionally, this dataset serves as a benchmark for researchers to develop improved technologies for similar tasks. Moreover, depending on the users, this dataset can serve multiple purposes in different levels of technology development, from counting objects to estimating litter coverage, as it provides both bounding box-based and pixel-based annotations.

## 1 Objective

The quantification of beach litter is a fundamental procedure in understanding the seriousness of pollution on beaches and addressing related issues. Hidaka et al. (2022) [2] developed a technique to identify artificial litter objects on beaches at the pixel level, but they were unable to identify plastic litter separately or count each object using the dataset [3] employed in the research. The Beach Plastic Litter Dataset version 1 (BePLi Dataset v1) enables the development of machine learning models that identify beach plastic litter at the instance or pixel levels. This BePLi Dataset v1 was created to facilitate the further advancement of the technique and to specialize in plastic litter quantity estimation, which have high demand from society.

## 2 Data Description

### 2.1 BePLi Dataset v1

**Fig. 1:**
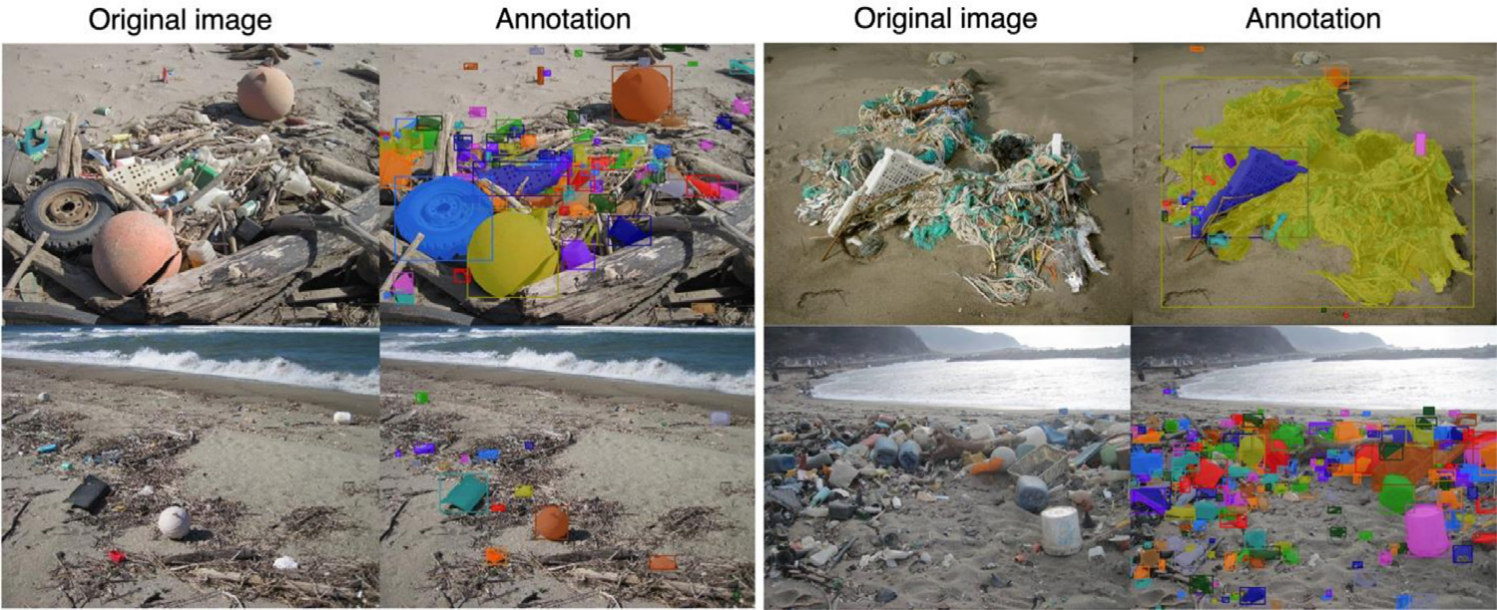
Annotation examples. Original images (left images in each column) and the visualization of the corresponding annotations for the plastic beach litter to the images (right images in each column).

The BePLi Dataset v1 comprises 3709 original image of beach plastic litter and corresponding instance segmentation annotations that were manually processed (Fig. 1). The images were taken at beaches along the entire coastline of Yamagata prefecture in Japan which is on the Japan Sea. The annotations for the object detection task were provided as JSON files compiled in the Microsoft Common Objects in Context (MS COCO) format (deliberately modified in part). All target objects were made of plastic and were categorized in a single object class called “plastic_litter”. The BePLi Dataset v1 was provided within the. “plastic_coco” directory, which contains the original images for training, validation, and testing as well as the corresponding MS COCO format-formatted JSON files. For the directory tree and description of this dataset, please refer to Table 1.

**Table 1:** Overview of the dataset directory structure and files.

Directory tree	Description
├— plastic_coco/	
│ ├— images/	
│ ├—├— original_images/	Directory containing original beach plastic litter PNG images for the combined dataset
│ ├—├— train/	Directory containing original beach plastic litter PNG images for the training set
│ ├—├— val/	Directory containing original beach plastic litter PNG images for the validation set
│ ├—├— test/	Directory containing original beach plastic litter PNG images for the test set
│ ├— annotations/	
│ ├—├— all_plastic_coco.json	MS COCO Object Detection Task formatted JSON file for the combined all dataset
│ ├—├— train.json	MS COCO Object Detection Task formatted JSON file for the training dataset
│ ├—├— val.json	MS COCO Object Detection Task formatted JSON file for the validation dataset
│ ├—├— test.json	MS COCO Object Detection Task formatted JSON file for the test dataset
└— README	Links for paper, disclaimer, and others.

### 2.2 plastic_coco directory

**Fig. 2:**
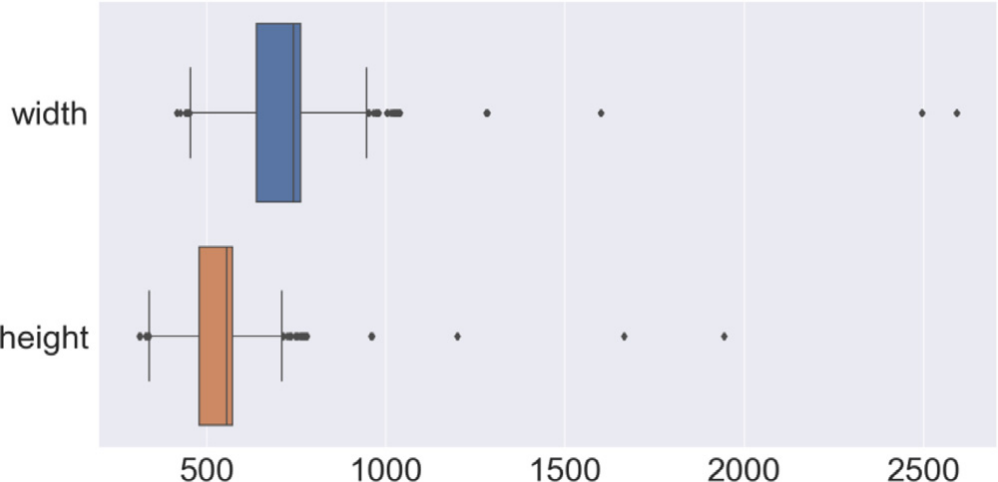
Boxplot of width and height of the original images. Both ends of the box are quartiles; the lines are at 1.5 times the quartile range, and the black dots represent outliers of this range.

The image directory consists of four subdirectories: original_images, train, val, and test, which contain 3709, 2226, 742, and 741, original PNG images, respectively. The images in each subdirectory are named sequentially, e.g., 001234.png, with the filenames corresponded to the “file_name” entry in the MS COCO-formatted annotation file described below. The resolution of the original images varied, with the median width and median height being 743 and 557 pixels, respectively as shown in Fig. 2. The aspect ratio of 97% of the images was 4:3.

The annotation directory included four files: all_plastic_coco.json, train.json, val.json, and test.json, which were formatted in the MS COCO Object Detection Task JSON file and partially modified. In the original MS COCO format, annotations for the single and crowd object were compiled in different data encodings, namely, “Polygon” and “Run-length encoding (RLE)” When segmentation for a single object was annotated, the entry of “iscrowd” was set to “0” in the “annotation” section and was encoded as a polygon. However, for crowded objects “iscrowd” was set to “1”, and the annotation was encoded as RLE encoding. However, because a polygon cannot express a hollowed-out object, such as a “tire”, the BePLi Dataset v1 used RLE encoding for the instance segmentation of a single object. Moreover, the “iscrowd” entry was intentionally set to “1”, and all the annotations were given for single objects.

**Fig. 3:**
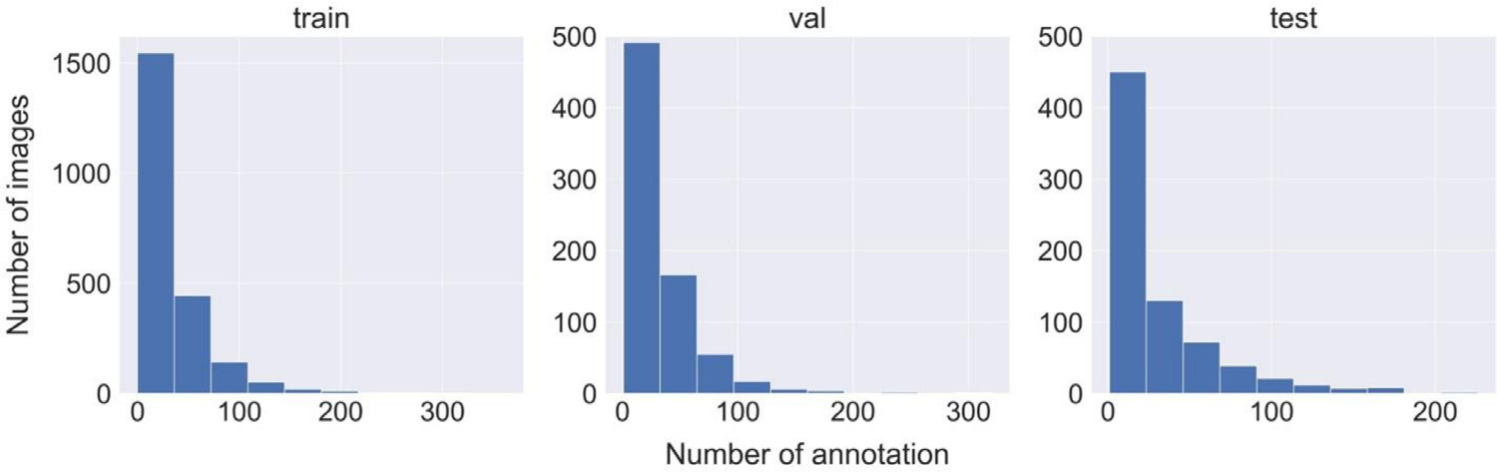
Histograms of the number of annotations in each image on BePLi Dataset v1.

**Fig. 4:**
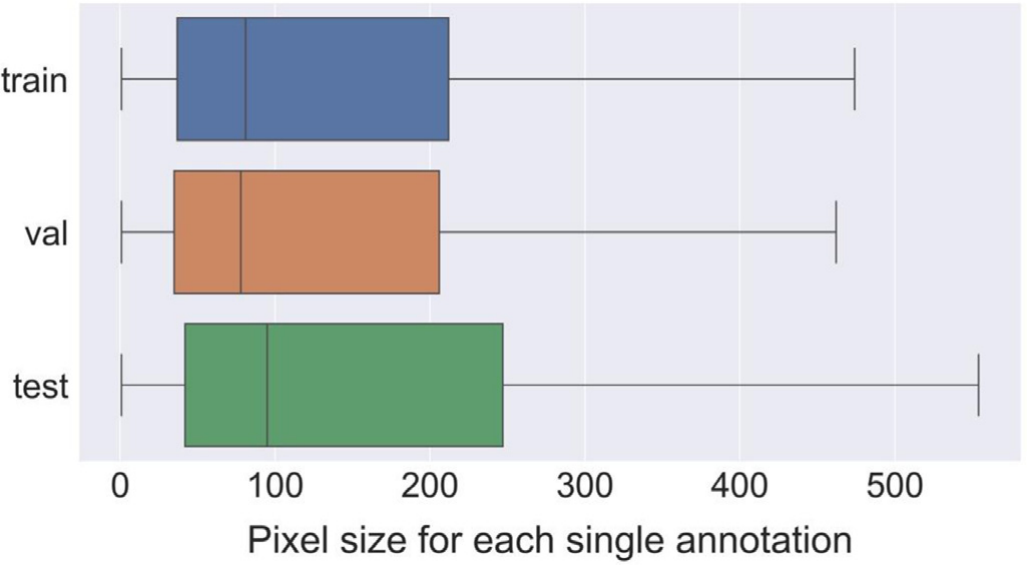
Boxplot of the pixel size distribution for each annotation for instance-segmentation on BePLi Dataset v1; the lines are at 1.5 times the quartile range. The outliers were removed from this plot.

The total number of annotations on the BePLi Dataset v1 was 119192, and the average annotation for each image was 32.14. Fig. 3 shows the number of annotations in each image in the training, validation, and test dataset. The pixel size distribution of each annotation, for instance segmentation, is shown in Fig. 4.

## 3 Experimental Design, Materials and Methods

The BePLi Dataset v1 was created using 3709 original beach images obtained from beaches located along the entire coast of Yamagata Prefecture, which is situated in the northern part of Japan (38°14′26″N, 140°21′48″E), with its coastline facing the Japan Sea, where a significant amount of litter drifts from marginal Asian countries. The images were obtained from beach litter monitoring programs that have been operated by the local government twice a year, during spring and autumn, since 2011, across 167 monitoring sites. The monitoring method, developed by the Non-Profit Organization Partnership Office, is visual-based, and details can be found in [4]. During the monitoring, observers recorded images of the beach and litter using standard consumer digital cameras from three (front, left, and right) or four (front, left, right, and back) directions and took extra close-up shots of the litter. The images were taken in different scenarios, such as sand beaches, rocky beaches, and tetrapods. All the images were pasted on monitoring records in Microsoft Excel files, from which 3709 images were extracted for the BePLi Dataset v1. The Excel files included locality information on where each image was taken, but this information cannot be made public due to an agreement between the local government and the dataset creators. To create the corresponding instance segmentation annotations for beach plastic litter, manual annotations were performed using Adobe Photoshop (Adobe Inc.), where segmentation was given for each single plastic litter object and saved as mask PNG files. The annotation was given for all objects made from plastics, such as PET bottles, containers, fishing gears, styrene foams, and fragmented plastics, and all annotations were classified under one category, “plastic_litter.” The first manual annotations were performed by an outsourcing company, after which researchers, engineers, and students conducted data checks and quality control. The acceptance of errors for the manual annotations on instance segmentation was ±3 pixels. Lastly, JSON files formatted in MS COCO (Object Detection Task) were created from the mask PNG files using Python libraries, such as Pycocotools 2.0 and Pillow 8.4, and running codes uniquely developed for the task. The JSON files were deliberately modified in part, as described in the data description section.

## Ethics Statements

Not applicable.

## CRediT Author Statement

**Mitsuko Hidaka:** Conceptualization, Methodology, Data quality control, Data curation, Formal analysis, Software, Writing – original draft; **Koshiro Murakami:** Data quality control, Formal analysis, Software, Writing – review and editing; **Kenta Koshidawa:** Data quality control, Formal analysis; **Shintaro Kawahara:** Data quality control, Formal analysis, Software, Writing – review and editing; **Daisuke Sugiyama:** Supervision, Software, Writing – review and editing; **Shin'ichiro Kako:** Supervision, Writing – review and editing; **Daisuke Matsuoka:** Project administration, Supervision, Writing – review and editing.

## Declaration of Competing Interest

The authors declare that they have no known competing financial interests or personal relationships that could have influence the work reported in this paper.

## Data Availability

BePLi Dataset v1: Beach Plastic Litter Dataset version 1 (Original data) (Seanoe).
